# Smart-ORF: a single-molecule method for accessing ribosome dynamics in both upstream and main open reading frames

**DOI:** 10.1093/nar/gkaa1185

**Published:** 2020-12-16

**Authors:** Anthony Gaba, Hongyun Wang, Trinisia Fortune, Xiaohui Qu

**Affiliations:** Molecular Biology Program, Memorial Sloan Kettering Cancer Center, New York, NY 10065, USA; Molecular Biology Program, Memorial Sloan Kettering Cancer Center, New York, NY 10065, USA; Molecular Biology Program, Memorial Sloan Kettering Cancer Center, New York, NY 10065, USA; Molecular Biology Program, Memorial Sloan Kettering Cancer Center, New York, NY 10065, USA

## Abstract

Upstream open reading frame (uORF) translation disrupts scanning 43S flux on mRNA and modulates main open reading frame (mORF) translation efficiency. Current tools, however, have limited access to ribosome dynamics in both upstream and main ORFs of an mRNA. Here, we develop a new two-color *in vitro* fluorescence assay, Smart-ORF, that monitors individual uORF and mORF translation events in real-time with single-molecule resolution. We demonstrate the utility of Smart-ORF by applying it to uORF-encoded arginine attenuator peptide (AAP)-mediated translational regulation. The method enabled quantification of uORF and mORF initiation efficiencies, 80S dwell time, polysome formation, and the correlation between uORF and mORF translation dynamics. Smart-ORF revealed that AAP-mediated 80S stalling in the uORF stimulates the uORF initiation efficiency and promotes clustering of slower uORF-translating ribosomes. This technology provides a new tool that can reveal previously uncharacterized dynamics of uORF-containing mRNA translation.

## INTRODUCTION

Eukaryotic translation initiation generally occurs through a scanning mechanism that begins with formation of the 43S pre-initiation complex (PIC), consisting of a 40S ribosomal subunit bound by a ternary complex (eukaryotic initiation factor (eIF) 2, GTP and methionyl initiator tRNA), eIF1, eIF1A, eIF3 and eIF5 (1). 43S attachment to mRNA is directed toward the 7-methylguanosine (m^7^G) cap at the mRNA 5′ end, from which the 43S scans the mRNA leader in a 3′ direction searching for a start codon. Start codon recognition stops the scanning 43S and leads to the release of eIFs, PIC structural rearrangements, and 60S subunit joining to form the 80S ribosome (2), which then proceeds to the peptide elongation stage. This canonical initiation pathway is regulated by a diverse range of regulatory elements (1), including upstream open reading frames (uORFs) (3). uORF start codons are located upstream of the main ORF (mORF) and their coding sequences can either remain in the 5′ leader or extend into the mORF (4). Bioinformatic estimates suggest that uORFs are present in as many as 50% of all eukaryotic mRNAs and ribosome footprint profiling studies detect initiation at a significant fraction of these uORFs (5–7). Genome-wide, uORF start codons generally contain poor initiation contexts that inefficiently capture scanning 43S, which will continue to scan toward the mORF (8). This flux of 43S scanning to a mORF is disrupted by 43S uORF start codon recognition and 80S uORF translation, which leads to down-regulation of mORF translation. In addition, uORF-mediated translational control can be affected by other *cis*- and *trans*-acting regulatory elements (4), thereby diversifying the dynamic regulation of mORF translation efficiency.

Kinetic measurements that track ribosome dynamics in both the uORF and mORF of an mRNA would enable insights into the influence of ribosome flux on mORF translation dynamics. To achieve these kinetic measurements, however, new techniques are needed. Studies of uORF-mediated translational control have thus far used genetic (9–11), structural (12), biochemical (4,11,13) and genomic (8,10) bulk approaches. Although these methods have allowed extensive uORF characterization, they are limited to ensemble averaging of signals from heterogeneous and asynchronous translation events. Bulk methods are therefore unable to access the translation dynamics of individual ribosomes. As a specialized technique for measuring the kinetics of molecular events, single-molecule methods have been successfully applied to studies of several eukaryotic translation processes, including eIF interactions in the absence of translation (14,15), IRES-mediated initiation (16), elongation (17–19), translation kinetics in live cells (20), and individual ribosome dynamics on single ORF-containing mRNA during active *in vitro* translation (21). Single-molecule methods for tracking individual eukaryotic ribosome dynamics of both uORF and mORF translation have not been reported.

We previously developed a single-molecule system that enables tracking of single ribosome translation kinetics during active translation of a single ORF (21). This approach is based on detection of fluorescently-labeled antibody binding to nascent N-terminal-tagged polypeptide during cell-free translation. Here we extend this one-color single-molecule assay to a two-color assay that allows simultaneous real-time detection of both nascent mORF- and uORF-encoded polypeptides from an individual mRNA. This single-molecule method is referred to hereafter as Smart-ORF (single-molecule analysis of ribosome dynamics in two open reading frames). To demonstrate Smart-ORF’s capability, we applied the approach to study translational control mediated by the uORF-encoded arginine attenuator peptide (AAP). The AAP belongs to a class of polypeptides, known as ribosome arrest peptides (22), that function in their nascent form and act from within the ribosome exit tunnel to arrest their own translation at either the elongation or termination step. In mammalian (23), fungal (13), plant (24) and viral (25) systems, uORF-encoded ribosome arrest peptides stall ribosomes to negatively regulate mORF translation for a variety of physiological processes. In fungi, the AAP stalls ribosomes at its encoding uORF’s termination codon to reduce synthesis of the mORF-encoded glutamine amidotransferase subunit of Arg-specific carbamoyl phosphate synthetase (26,27). By stalling ribosomes in response to Arg surplus, the AAP reduces Arg biosynthesis and helps to control Arg homeostasis (26). Although AAP-mediated translational control studies have used genetic (27), biochemical (13,28,29), and structural (12) approaches, translation kinetics of AAP-mediated regulation remains incompletely understood. Our application of Smart-ORF to the AAP system allowed us to detect, resolve, and quantify dynamics of individual mORF- and uORF-translating ribosomes. The observed kinetics reveal that the AAP regulates ribosome occupancy of the uORF through various modes, which collectively modulate ribosome flux and control mORF translation.

## MATERIALS AND METHODS

### Plasmids, DNA templates and RNA synthesis

*AAP_FLAG_-LUC_HA_* DNA templates were generated from plasmid derivatives of pAG101(13). A synthetic DNA fragment (GenScript) with 5′ NcoI and 3′ KasI restriction sites and a 10x HA tandem repeat coding sequence fused in-frame to the 5′-end of the firefly LUC ORF, was cloned into the corresponding restriction sites of pAG101. The modified pAG101 was then digested with BglII and NcoI and this region was replaced with a BglII/NcoI-flanking synthetic DNA fragment (GenScript) containing in-frame a 3x FLAG tag, 42x CAA repeat, and the *S. cerevisiae* AAP coding sequence followed by the *CPA1* intercistronic region. The resulting plasmid, pAG143, was mutated to introduce an Asp to Asn codon change at codon 13 of the *CPA1* uORF by using primers AG100 (5′-CACCTGCCAAAACTACATATC-3′) and AG101 (5′-TATTGAGAGTTCGATAAGC-3′) and the Q5 Site-Directed Mutagenesis kit (New England Biolabs) to generate pAG144. The uORF initiation context in pAG143 and pAG144 was improved by using primers AG109 (5′-AAAATGTTCGACTACAAAGAC-3′) and AG110 (5′-TCAATTTTTTTATTTCAAATCTGCAAAAAG-3′) with the Q5 Site-Directed Mutagenesis kit (New England Biolabs) to generate pAG146 and pAG147, respectively.

Polyadenylated *AAP_FLAG_-LUC_HA_* mRNA was synthesized with the MEGAscript T7 Kit (Ambion) according to the manufacturer's protocol and with PCR product templates generated with the Q5 High-Fidelity PCR Kit (New England Biolabs) and primers AG105 (5′- CATTAGGAAGCAGCCCAGTAGTAG-3′) and AG107 (5′-CTTCCGGCTCGTATGTTGTGTG-3′). Polyadenylated mRNA was 5′ capped using the Vaccinia Capping System (New England Biolabs) and was 3′ biotinylated using the Pierce RNA 3′ End Biotinylation Kit (Thermo Scientific). For Renilla control mRNA, DNA template preparation and mRNA synthesis were as previously described (30). All mRNAs were phenol-chloroform extracted and subsequently purified with the Direct-Zol RNA kit (Zymo Research), from which mRNA was eluted in water. Integrity and concentration of mRNA was assessed by denaturing (8 M urea) 5% acrylamide gel electrophoresis and SYBR green II RNA staining.

### Cell extract and Cell-Free translation

Translation extract derived from *S. cerevisiae* strain YAS1874 (*MAT***a***MAK10::URA3 PEP4::HIS3 prb1 prc1 ade2 trp1 his3 ura3*) (31) was prepared as described previously (32) with the following modifications: (i) yeast cultures were grown until OD_600_ ≈ 2.0, (ii) buffer A was pH 7.4, (iii) buffer for breaking cells was supplemented with a protease inhibitor cocktail for fungal and yeast cells (Sigma), (iv) lysates were clarified by two 10 min centrifugations at 16 000 rpm in a SS-34 rotor followed by a 10 min centrifugation at 14 000 rpm in a microfuge and (v) small molecules were removed from the clarified lysates with Zeba Desalt Spin Columns (Pierce), used according to the manufacturer's protocol and preequilibrated with buffer A. Consistency in extract preparation, reagent storage, and experimental execution is critical for suppressing experimental variation with this single-molecule assay (33).

Bulk *in vitro* translation reaction conditions were essentially as described previously (13). Briefly, 10 μl translation reactions containing 5 ng *AAP_FLAG_-LUC_HA_* mRNA and 0.5 ng *Renilla* luciferase mRNA were incubated at 25°C for 30 min and terminated by freezing in liquid nitrogen. Ice-thawed reaction mixtures were diluted with an equal volume of 2x passive lysis buffer (Promega) and 8 μl aliquots of the diluted reactions were used to measure firefly and Renilla luciferase enzyme production with a dual luciferase reporter assay (Promega), used according to the manufacturer's protocol except that 50 μl of Luciferase Assay Reagent II and 50 μl of Stop & Glo reagent were used. Luminescence was measured with a GloMax 96 Microplate Luminometer. The firefly luciferase activity levels were normalized to the levels of Renilla luciferase activity, and were then normalized to the luminescence from reactions with *WT-AAP_FLAG_-LUC_HA_* mRNA and 10 μM Arg. Translation reaction mixtures for single-molecule experiments were assembled as with bulk reactions, except that the mixtures were not supplemented with mRNA and contained 10% glycerol, 10 ng/μl Cy3-anti-FLAG (Sigma, A9594), and 1.125 ng/μl Alexa Flour 647-anti-HA (Santa Cruz, sc-7392 AF647), unless specified otherwise. Cy3-anti-FLAG has a labeling ratio of 2–7 fluorophores per antibody as specified by the manufacturer's product manual. Alexa Flour 647-anti-HA has a labeling ratio of 1–8 fluorophores per antibody (mean value: 3) as determined by counting fluorophore photobleaching steps (34). All translation reactions contained 4.0 mM Mg^2+^ and 160 mM K^+^.

### Single-Molecule methods and data analysis

Single-molecule detection chambers were assembled as described previously (21). Individual flow channels were incubated with 10 μl of 0.2 μg/μl streptavidin (Thermo Scientific) for 10 min followed by three washes, each with 15 μl of T50+RNasin buffer (20 mM Tris–HCl (pH 7.0), 50 mM NaCl, 0.2 units/μl RNasin (Promega)). Channels were then flushed with 20–60 ng of 3′-end biotinylated mRNA, incubated for 15 min, and washed with three 15 μl flushes of T50+RNasin buffer. Channels were then flushed with two sequential 15 μl additions of 0.5 nM Cy3-CAA oligo (5′-CAACAACAACAACAACAA-3′) and incubated for 5 min to fluorescently label immobilized mRNA. Unbound Cy3-CAA oligo was removed by three 15 μl washes with T50+RNasin buffer and fluorescence was imaged to assess relative mRNA density in channels. Cy3-CAA oligo annealed to mRNA was removed by flushing channels with 12 U of DNase I in 1X reaction buffer (10 mM Tris–HCl (pH 7.6), 2.5 mM MgCl_2_, 0.5 mM CaCl_2_) and incubating for 5 min. Channels were then flushed three times with 15 μl of T50+RNasin buffer per flush followed by imaging of the detection surface to ensure that the Cy3-CAA oligo was completely removed. Channels were then flushed four times with 15 μl of channel wash buffer (4.0 mM MgOAc, 160 mM KOAc, 35 mM HEPES, 2 mM DTT) per flush. Translation reaction mixtures (20 μl) were delivered to chambers with a microfluidic-adapted Harvard Apparatus syringe pump at a speed of 150 μl/min. All flow chamber incubations were performed at 25°C.

Objective-type TIRF imaging was carried out using an Olympus IX83 inverted microscope equipped with a 100× oil immersion objective (N.A. 1.49), 100 mW 532 and 640 nm lasers with adjustable outputs, CellTIRF illuminator, Andor iXon Ultra 897 EMCCD, Chroma 532/640/25 excitation filter, Semrock R405/488/532/635 dichroic, and Semrock NF03-405/488/532/635E-25 emission filter. Laser illumination intensities at the objective for the 532 and 640 nm lasers were 10 and 4 μW, respectively. Imaging conditions were previously described (21). The data was recorded as a kinetic series at a speed of 2 s/frame for a total of 60 min. This time resolution was chosen based on the observed cap-dependent translation kinetics in this translation extract (21). A faster time resolution may be applied to systems with faster translation kinetics. Movies were initially analyzed with custom written Matlab codes to correct for drift and background. Cy3-αFLAG and AF647-αHA bound in each frame were identified based on their respective fluorescence intensities. Positions of bound antibodies were determined with pixel-level accuracy by using local maxima and intensity threshold levels. Cy3-αFLAG and AF647-αHA binding events at specific locations were analyzed as trajectories that were generated by connecting the individual Cy3 and AF647 fluorescence intensities from all frames in a movie. Fluorescence intensity traces were filtered with a nonlinear forward–backward filter (35) to reduce trajectory noise and distortion. A custom step-detection algorithm (36) written in Python was used to extract the timing of Cy3-αFLAG and AF647-αHA binding and dissociation from trajectories.

To exclude from our kinetic analyses fluorescence changes that were due to diffusing antibodies, a Cy3-αFLAG and AF647-αHA binding dwell time cut-off of 20 s was applied for all trajectory analyses. The first arrival time was defined as the time from translation mixture addition to a trajectory's first fluorescence step increase. The number of antibody binding events per trajectory was calculated as the total number of fluorescence step increases in each trajectory. Lag-times between Cy3-αFLAG binding events in trajectories were calculated by measuring the time intervals between neighboring Cy3 fluorescence step increases. For isolated Cy3-αFLAG binding events, dwell time was calculated using the threshold approach as previously described (21). For clustered Cy3-αFLAG binding events, the average individual antibody dwell time in a cluster was measured using the intensity integration approach. First, the average fluorescence intensity of individual Cy3-αFLAG antibodies were determined from the Gaussian distribution of isolated Cy3-αFLAG fluorescence intensities in each data set. The average dwell time of the individual antibodies in clusters was then calculated by dividing the area intensity integration of a cluster by the product of the total number of fluorescence step increases in a cluster and the calculated average intensity of individual Cy3-αFLAG antibodies. For both approaches, binding events in which Cy3-αFLAG did not yet dissociate by the end of data acquisition were excluded from dwell time analyses since the dissociation time could not be determined. The standard error of all mean and median calculations was determined by bootstrapping as previously described (37).

## RESULTS

### Smart-ORF: a two-color single-molecule assay for simultaneous tracking of individual uORF and mORF translation events

**Figure 1.**
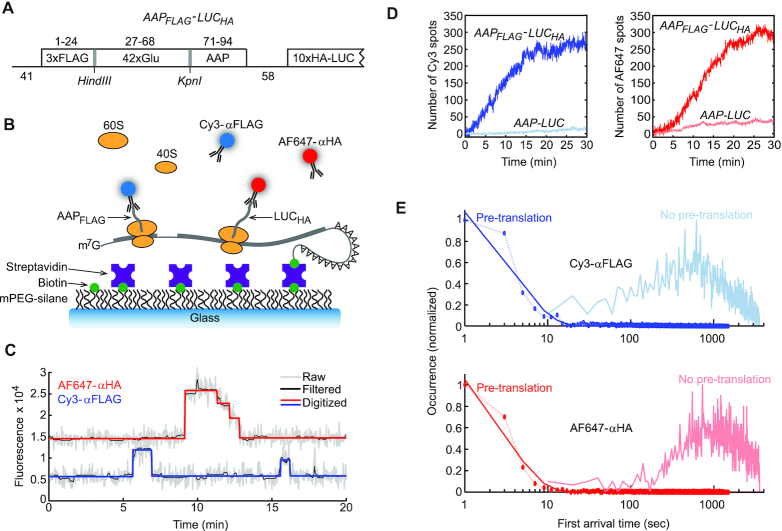
Smart-ORF cell-free system for imaging both uORF and mORF translation on single mRNA molecules. (**A**) Construct map of *AAP_FLAG_*-*LUC_HA_* mRNA. The *AAP_FLAG_* uORF encodes a polypeptide composed of a 3xFLAG tag, 42xGlu repeat, and the *S. cerevisiae* AAP. The mORF encodes a polypeptide composed of a 10xHA tag and firefly luciferase. The AAP_FLAG_ residues are indicated above their coding regions. The nucleotide lengths of the region upstream of the uORF and the intercistronic region are indicated. Sequences of *AAP_FLAG_*-*LUC_HA_* genes are shown in Supplementary Figure S1b. (**B**) Schematic of the single-molecule assay. 3′-end biotinylated mRNA molecules are end-tethered to a PEGylated detection surface via biotin-streptavidin interactions. *S. cerevisiae* translation mixture supplemented with Cy3-αFLAG and AF647-αHA is flowed into the detection chamber and uORF and mORF translation dynamics on single mRNA molecules are tracked by Cy3-αFLAG and AF647-αHA binding to nascent AAP_FLAG_ and LUC_HA_, respectively. (**C**) Representative trajectories for the translation dynamics on a single *AAP_FLAG_-LUC_HA_* mRNA. Upper and lower trajectories are for AF647-αHA (red) and Cy3-αFLAG (blue) binding kinetics, respectively. Raw, filtered, and digitized data are shown as indicated. (**D**) Representative results for the number of Cy3 (blue) and AF647 (red) spots in a field of view for the translation of *WT-AAP_FLAG_*-*LUC_HA_* or tag-lacking *WT-AAP-LUC* mRNA. The antibody binding with these two mRNAs represents specific binding to nascent peptide and nonspecific binding to single-molecule detection surface, respectively. The ratios of plateau values for specific to nonspecific antibody binding were ∼9–14 (Cy3-αFLAG) and ∼5–9 (AF647-αHA), respectively. (**E**) The distribution of the first arrival time of Cy3-αFLAG (blue) and AF647-αHA (red) binding to *WT-AAP_FLAG_*-*LUC_HA_* mRNA with (darker colors) or without (lighter colors) pre-translation. To determine the time constant of antibody binding to pre-generated nascent peptides, the histograms with pre-translation were fit to the single-exponential distribution $f( x ) = a \cdot {e^{ - \frac{x}{{{\tau _1}}}}}$, which yielded τ_1_ = 4.0 ± 0.4 (s.e.) s and 3.1 ± 0.2 (s.e.) s for Cy3-αFLAG and AF647-αHA, respectively. The x-axes are shown in log scale for better visibility of first arrival time with the pre-translation condition. These two plots are shown again in Supplementary Figure S1c with the x-axes in a linear scale.

To track individual ORF translation events during active translation, we recently developed a cell-free system wherein fluorophore conjugated antibodies bind to N-terminal epitopes on nascent polypeptides translated from monocistronic transcripts (21). Based on this previous assay, we developed Smart-ORF by using two antibody-epitope pairs and two-color fluorescence imaging. To assess the method's capability, we applied Smart-ORF to analyze ribosome dynamics in AAP-mediated translational control. Specifically, bicistronic genes (*AAP_FLAG_-LUC_HA_*) were designed to contain a mORF encoding firefly luciferase with an N-terminal 10xHA tag (LUC_HA_) and a uORF encoding a polypeptide consisting of an N-terminal 3xFLAG tag, an internal 42x glutamine repeat region, and a C-terminal AAP (AAP_FLAG_) (Figure 1A). Based on a ribosome exit tunnel length of approximately 35 amino acids (38,39), the uORF-encoded glutamine repeat serves as a spacer to allow N-terminal 3xFLAG tag exposure from the ribosome prior to translation of the C-terminal AAP. The spacer region was specifically encoded by the glutamine codon, CAA, because CAA repeat sequences do not introduce cognate initiation sites, near-cognate initiation sites, or stable secondary structures (40,41) (Supplementary Figure S1a, b). All mRNAs used in this study were 5′-end m^7^G capped and 3′-end biotinylated.

*AAP_FLAG_-LUC_HA_* mRNAs were immobilized to streptavidin-coated detection surfaces via the 3′ biotin (Figure 1B). Flow chambers were then flushed with cell-free translation mixtures consisting of *Saccharomyces cerevisiae* translation extract, 67 nM Cy3-labeled anti-FLAG antibody (Cy3-αFLAG), 7.5 nM Alexa Fluor 647-labeled anti-HA antibody (AF647-αHA), and either 10 μM (low) or 2 mM (high) Arg. During *AAP_FLAG_-LUC_HA_* mRNA translation, the FLAG and HA tags emerge from the ribosome exit tunnel and become accessible to interact with Cy3-αFLAG and AF647-αHA, respectively (Figure 1B). The interactions between the fluorescent antibodies and nascent peptides were imaged by a total internal reflection fluorescence (TIRF) microscope at single-molecule resolution and were recorded in time-lapse movies. Cy3-αFLAG and AF647-αHA interactions with individual nascent AAP_FLAG_ and LUC_HA_ peptides, respectively, were analyzed as trajectories showing Cy3 and AF647 fluorescence changes for individual mRNA molecules (Figure 1C). We demonstrated previously that Cy3-αFLAG photobleaching is negligible in our single-molecule conditions, which enables full-scale kinetic analysis (21). In contrast, the AF647 trajectories showed significant photobleaching over the timescale of individual translation events (Figure 1C), limiting dwell-time analysis. For example, in the red trajectory in Figure 1C, the binding of an individual AF647-αHA antibody was followed by successive photobleaching of individual AF647 dyes on the multiple-labeled αHA antibody. Accordingly, only the onsets of AF647-αHA binding events were analyzed.

The time-resolved kinetics of antibody binding amount per field of view showed that accumulation of bound Cy3-αFLAG and AF647-αHA began approximately 1 and 3 min, respectively, after translation mixtures were flushed into *AAP_FLAG_-LUC_HA_* mRNA-containing chambers and continued accumulating for ∼20–30 min until reaching a plateau (Figure 1D). The kinetics we observed for Cy3-αFLAG and AF647-αHA binding accumulation are similar to the published kinetics of toeprint signal appearances at the *Neurospora crassa* AAP-encoding uORF and luciferase-encoding mORF, respectively, on *AAP-LUC* mRNA in bulk cell-free translation reactions (42). Furthermore, with single-molecule translation of *AAP-LUC* mRNA, which encodes AAP and firefly luciferase but lacks FLAG and HA tags, the accumulation of bound Cy3-αFLAG and AF647-αHA appeared linear and was ∼6–10-fold lower than the plateau values for *AAP_FLAG_-LUC_HA_* mRNA translation (Figure 1D). These ratios of antibody binding plateau values, however, do not directly reflect the ratios of specific vs. nonspecific total binding numbers in experiments because specific and nonspecific binding kinetics are distinct. Specifically, the ratio of specific to nonspecific binding numbers is in the range of 50–65 for Cy3-αFLAG binding and 13–17 for AF647-αHA binding for all experimental conditions. Accordingly, the amounts of specific antibody binding to translated AAP_FLAG_ and LUC_HA_ were well above the levels of nonspecific antibody binding in our system.

The kinetics of Cy3-αFLAG and AF647-αHA binding to translated AAP_FLAG_ and LUC_HA_, respectively, were assessed following our previously established approach (21). Specifically, chambers with *AAP_FLAG_-LUC_HA_* mRNA were flushed with antibody-lacking translation mixture, incubated for 20 min to generate nascent AAP_FLAG_ and LUC_HA_ polypeptides, and then flushed with a fresh translation mixture that contained antibodies. These flushed Cy3-αFLAG and AF647-αHA showed first arrival time constants of 4.0 ± 0.4 (s.e.) s and 3.1 ± 0.2 (s.e.) s, respectively, for antibody binding to the pre-generated nascent AAP_FLAG_ and LUC_HA_ peptides. In contrast, without pre-translation, the first arrival binding of Cy3-αFLAG and AF647-αHA was approximately 60 and 150 s, respectively (Figure 1E and Supplementary Figure S1c). The significantly faster antibody first arrival times in chambers with pre-translation indicated that the Cy3-αFLAG and AF647-αHA binding rates to AAP_FLAG_ and LUC_HA_, respectively, were not rate-limiting for detection of initiation kinetics.

### Smart-ORF detects AAP_FLAG_-mediated Arg-induced regulation of mORF translation on *AAP_FLAG_-LUC_HA_* mRNA

**Figure 2.**
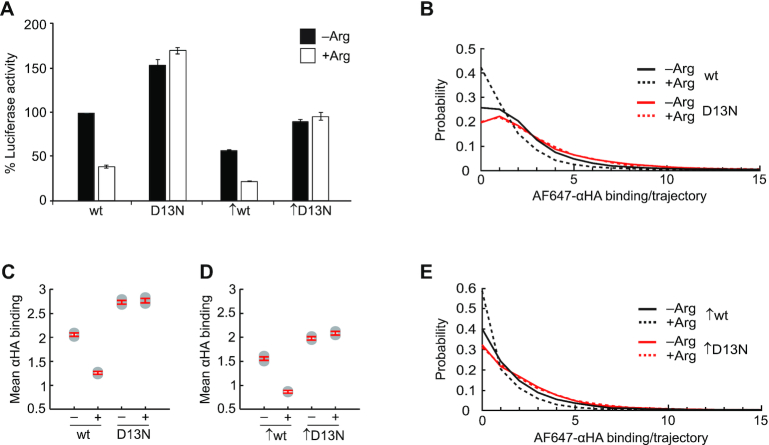
Bulk and Smart-ORF methods detect uORF-mediated regulation of mORF translation in response to Arg. All experiments were performed using four *AAP_FLAG_*-*LUC_HA_* mRNAs with uORFs encoding either the wild-type (wt) or D13N AAP_FLAG_ polypeptide and containing either the native or improved (↑) initiation context. The translation mixtures were supplemented with either 10 μM (–) or 2 mM (+) Arg. The same notations for mRNA identities and Arg conditions are applied to all subsequent figures. (**A**) Relative levels of firefly luciferase production in bulk cell-free translation reactions. All firefly luminescence readings were normalized to the wt –Arg condition and to the activity of an internal control Renilla luciferase (Materials and Methods). Mean values and standard deviations from two independent translation reactions are given. (**B**, **C**) Smart-ORF-derived probability distributions (**B**) and mean values (**C**) of AF647-αHA binding number per trajectory with native-context *AAP_FLAG_*-*LUC_HA_* mRNAs. Mean ± SEM: 2.05 ± 0.03 (wt –Arg), 1.26 ± 0.02 (wt +Arg), 2.75 ± 0.03 (D13N –Arg), 2.76 ± 0.03 (D13N +Arg). Numbers of trajectories analyzed: wt –Arg, *n* = 7613; wt +Arg, *n* = 9563; D13N –Arg, *n* = 9258; D13N +Arg, *n* = 5493. (D, E) Smart-ORF-derived mean values (**D**) and probability distributions (**E**) of AF647-αHA binding number per trajectory with improved-context *AAP_FLAG_*-*LUC_HA_* mRNAs. Mean ± SEM: 1.55 ± 0.03 (↑wt –Arg), 0.87 ± 0.01 (↑wt +Arg), 1.97 ± 0.02 (↑D13N –Arg), 2.09 ± 0.03 (↑D13N +Arg). Numbers of trajectories analyzed: ↑wt –Arg, *n* = 5747; ↑wt +Arg, *n* = 16 658; ↑D13N –Arg, *n* = 9672; ↑D13N +Arg, *n* = 9821. The S.E. of median and mean values in all figures were determined by a bootstrapping approach (Materials and Methods).

Previous bulk studies of AAP-mediated translational control used reporter *AAP-LUC* mRNAs composed of the uORF-containing 5′-leader region from either *CPA1* (*S. cerevisiae*) or *arg-2* (*N. crassa*) mRNA and a mORF encoding firefly luciferase (13,43,44). These studies showed that the AAP response to high Arg concentration causes ribosome stalling at the uORF termination codon and reduces translation at the downstream mORF. To test whether the *AAP_FLAG_-LUC_HA_* reporter mRNA preserved uORF-encoded AAP regulatory function, we measured luciferase activity in bulk cell-free translation reactions containing wild-type or D13N mutant *AAP_FLAG_-LUC_HA_* mRNA and either a low or high Arg concentration (Figure 2A). *D13N-AAP_FLAG_-LUC_HA_* mRNA contained an AAP missense mutation, D13N (Supplementary Figure S1b), that was previously found to eliminate Arg-induced translational regulation and ribosome stalling in bulk studies (13,27). Our bulk measurements showed that *WT-AAP_FLAG_-LUC_HA_* (wild-type AAP) mRNA translation reduced luciferase activity ∼2.5-fold in response to high Arg (Figure 2A). This Arg effect was absent with *D13N-AAP_FLAG_-LUC_HA_* mRNA translation (Figure 2A). We also observed that the wild-type AAP_FLAG_ inhibited *LUC_HA_* expression in low Arg relative to the D13N control (Figure 2A), indicating that the low Arg level in the cell-free translation reactions was sufficient for a partial regulatory response. These results are consistent with the AAP-mediated and Arg-specific effects observed previously in bulk cell-free translation reactions (13,42). Thus, in bulk cell-free translation reactions with *AAP_FLAG_-LUC_HA_* mRNA, the wild-type AAP_FLAG_ functions to negatively regulate *LUC_HA_* mORF translation in response to high Arg.

Our single-molecule approach enables tracking of individual translating ribosomes at the *LUC_HA_* mORF through the detection of AF647-αHA binding. To determine if the Smart-ORF method detects AAP_FLAG_-mediated Arg-induced regulation of *LUC_HA_* translation, we analyzed the distributions of AF647-αHA binding amount per trajectory for single-molecule translation of *WT-AAP_FLAG_-LUC_HA_* or *D13N-AAP_FLAG_-LUC_HA_* mRNA in the presence of either low or high Arg (Figure 2B). The amount of AF647-αHA binding per trajectory was reduced by high Arg for *WT-AAP_FLAG_-LUC_HA_* mRNA (mean binding number: 2.05 ± 0.03 [–Arg] versus 1.26 ± 0.02 [+Arg]), but not for *D13N-AAP_FLAG_-LUC_HA_* mRNA (mean binding number: 2.75 ± 0.03 [–Arg] versus 2.76 ± 0.03 [+Arg]) (Figure 2C). Furthermore, single-molecule translation of *D13N-AAP_FLAG_-LUC_HA_* mRNA showed increased AF647-αHA binding amounts per trajectory compared to those amounts with *WT-AAP_FLAG_-LUC_HA_* mRNA (Figure 2C). These results show consistent regulation of *LUC*_HA_ translation with Smart-ORF (Figure 2B, C) and bulk (Figure 2A) approaches. This regulation is also consistent with previous bulk measurements of AAP-mediated regulation *in vitro* and *in vivo* (13,27,28,42). Wild-type AAP_FLAG_ in the Smart-ORF system, therefore, appeared to retain the AAP’s ability to negatively regulate mORF translation in response to high Arg.

To further confirm AAP_FLAG_ regulation of *LUC_HA_* mORF translation in response to Arg, we tested a set of mutated *AAP_FLAG_-LUC_HA_* mRNAs in which the inefficient *AAP_FLAG_* uORF initiation context of the *CPA1* uORF is changed to a more efficient *GCN4* uORF1-like initiation context (43) (Supplementary Figure S1b). Regardless of the Arg concentration and D13N mutation, the initiation context-improved *AAP_FLAG_-LUC_HA_* mRNAs showed reduced mORF translation in both bulk (Figure 2A) and Smart-ORF (Figure 2D, E) systems compared to the corresponding measurements with native-context mRNAs (Figure 2A–C). These observations confirm previous bulk studies indicating that improved uORF initiation efficiency has a general effect of downregulating mORF translation (28,42). Importantly, bulk and Smart-ORF approaches with context-improved mRNAs showed Arg-induced negative regulation of mORF translation with the wild-type, but not D13N, *AAP_FLAG_* uORF (Figure 2A, D, E). Collectively, these results indicate that *AAP_FLAG_-LUC_HA_* mRNA single-molecule translation preserved AAP function to regulate mORF translation and allowed Smart-ORF analysis of this uORF-mediated regulation.

### Regulated durations of individual and clustered ribosomes in the *AAP_FLAG_* uORF are quantified by Smart-ORF

**Figure 3.**
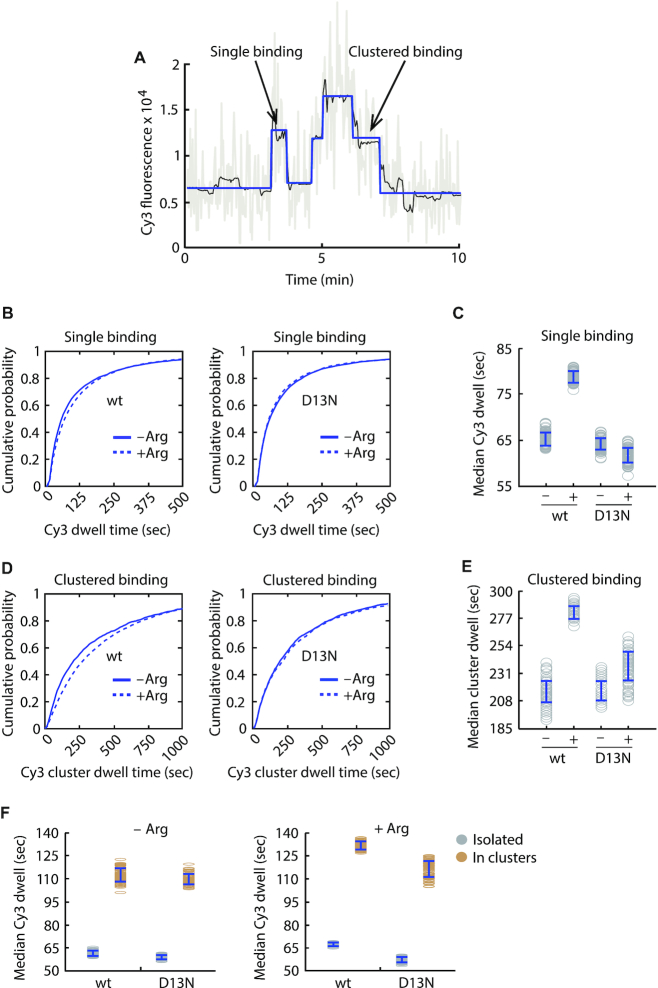
Smart-ORF resolves Arg-induced ribosome dwells in the native-context *AAP_FLAG_* uORF. (**A**) Representative single and clustered Cy3-αFLAG binding events in a Cy3 fluorescence trajectory showing uORF translation dynamics on a single *AAP_FLAG_-LUC_HA_* mRNA. Trajectory line colors are as described in Figure 1C. (B, C) Cumulative probability distributions (**B**) and median values (**C**) of Cy3-αFLAG dwell times for single binding events. Median and S.E. (s): 65 ± 1 (wt –Arg), 79 ± 1 (wt +Arg), 64 ± 1 (D13N –Arg), 62 ± 2 (D13N +Arg). Numbers of single binding events analyzed: wt –Arg, *n* = 5913; wt +Arg, *n* = 12 765; D13N –Arg, *n* = 7493; D13N +Arg, *n* = 3934. (D, E) Cumulative probability distributions (**D**) and median values (**E**) of clustered Cy3-αFLAG binding dwell times. Median and S.E. (s): 216 ± 9 (wt –Arg), 282 ± 5 (wt +Arg), 216 ± 8 (D13N –Arg), 237 ± 12 (D13N +Arg). Numbers of binding clusters analyzed: wt –Arg, *n* = 1580; wt +Arg, *n* = 5750; D13N –Arg, *n* = 2201; D13N +Arg, *n* = 952. The dwell times in (B)–(E) were analyzed using the threshold approach (Materials and Methods). (**F**) Median dwell times of individual Cy3-αFLAG from single (gray) and clustered (light brown) binding events. The single and clustered binding events in (B)–(E) were re-analyzed using the intensity integration approach (Materials and Methods). Single binding median dwell and S.E. (s): 62 ± 1 (wt –Arg), 67 ± 1 (wt +Arg), 59 ± 1 (D13N –Arg), 57 ± 1 (D13N +Arg). In cluster binding median dwell and S.E. (s): 113 ± 4 (wt –Arg), 131 ± 2 (wt +Arg), 109 ± 3 (D13N –Arg), 116 ± 5 (D13N +Arg). The numbers of binding events analyzed are the same as in (B)–(E).

Previous studies are consistent with a mechanism in which AAP-mediated ribosome arrest increases ribosome occupancy of AAP-encoding uORFs (13,42). However, little is known about the uORF occupancy duration of these regulated ribosomes. We previously demonstrated with our one-color single-molecule system that Cy3-αFLAG binding to nascent N-terminal 3xFLAG tag is rapid, as observed with Smart-ORF (Figure 1E and Supplementary Figure S1c), and that Cy3-αFLAG dissociation is a faithful tracker of ribosome dissociation from mRNA (21). The dwell time of Cy3-αFLAG binding to the uORF-encoded AAP_FLAG_, therefore, enables Smart-ORF to measure individual ribosome durations in the *AAP_FLAG_* uORF. The dwell time distributions of single isolated Cy3-αFLAG binding events (Figure 3A) showed that high Arg increased these dwell times by 14 s (median 65 ± 1 s [–Arg] versus 79 ± 1 s [+Arg]) for native-context *WT-AAP_FLAG_-LUC_HA_* mRNA (Figure 3B, C and Supplementary Figure S2a) and by 25 s (median 60 ± 1 s [–Arg] versus 85 ± 1 s [+Arg]) for context-improved *WT-AAP_FLAG_-LUC_HA_* mRNA (Supplementary Figures S2b and S3a,b). High Arg did not increase the dwell time of Cy3-αFLAG single binding events for *D13N-AAP_FLAG_-LUC_HA_* mRNAs with either the native-context (median 64 ± 1 s [–Arg] versus 62 ± 2 s [+Arg]; Figure 3B, C and Supplementary Figure S2a) or improved-context (median 70 ± 1 s [–Arg] versus 66 ± 1 s [+Arg]; Supplementary Figures S2b and S3a, b). These observations indicate that in response to high Arg, the wild-type AAP_FLAG_ but not the D13N AAP_FLAG_ mediates stalling of single ribosomes in the *AAP_FLAG_* uORF.

Cy3 trajectories also displayed clustered antibody binding events (Figure 3A), which represent translation of the *AAP_FLAG_* uORF by two or more ribosomes that are closely spaced in the uORF and are each associated with a nascent AAP_FLAG_-Cy3-αFLAG complex. Analysis of the Cy3-αFLAG clustered dwell time distributions showed that high Arg increased these dwells by 66 sec (216 ± 9 s [–Arg] versus 282 ± 5 s [+Arg]) with *WT-AAP_FLAG_-LUC_HA_* mRNA but did not significantly change the clustered dwells with *D13N-AAP_FLAG_-LUC_HA_* mRNA (median 216 ± 8 s [–Arg] versus 237 ± 12 s [+Arg]) (Figure 3D, E and Supplementary Figure S2c). Consistently, high Arg increased the clustered Cy3-αFLAG binding dwell time by 151 s (median 153 ± 6 s [–Arg] versus 304 ± 5 s [+Arg]) with the improved-context *WT-AAP_FLAG_-LUC_HA_* mRNA but did not have a significant effect with the improved-context *D13N-AAP_FLAG_-LUC_HA_* mRNA (median 224 ± 9 s [–Arg] versus 189 ± 5 s [+Arg]) (Supplementary Figures S2d and S3c, d). Taken together, these results indicate that the durations of both isolated and clustered ribosomes in the *AAP*_FLAG_ uORF were increased by the AAP_FLAG_ response to high Arg.

Notably, the Cy3-αFLAG binding dwell time measurements showed that the AAP_FLAG_-mediated response to high Arg increased single ribosome dwells by ∼22% and ∼42% for native and improved context mRNAs, respectively, but the corresponding increases for ribosome clusters were significantly greater and increased by ∼31% and ∼99%, respectively. One possible explanation for this observation is that ribosome clustering in the uORF caused slower translation of the uORF. This regulation could occur in addition to AAP-mediated single ribosome pausing. To investigate this potential cluster-specific effect on individual ribosomes, we sought to measure the dwell times of individual Cy3-αFLAG binding events in clusters and compare them to the dwell times of single isolated Cy3-αFLAG binding events. In the above dwell time analysis of single ribosomes, we computationally determined the beginning and ending of single antibody binding events from fluorescence intensity changes in trajectories (Materials and Methods). However, this threshold-based approach is unreliable when applied to clustered ribosome binding due to the kinetic complexity of these binding events. We therefore tested an alternative approach to derive the average dwell time of the individual Cy3-αFLAG binding events in clusters using intensity integration (Materials and Methods). As a control, we measured the single isolated Cy3-αFLAG binding events with both threshold- and intensity integration-based approaches and observed similar dwell time trends (Supplementary Figure S3e), validating the intensity integration dwell time analysis approach. Using the intensity integration approach, we found that the individual ribosome dwells in clustered binding events were significantly increased by ∼83–102% relative to the dwell times of single isolated ribosomes for all native-context mRNA conditions, regardless of D13N mutation and Arg concentration (Figure 3F). A similar cluster effect was observed with context-improved *AAP_FLAG_-LUC_HA_* mRNAs, which showed that clustering increased individual ribosome dwells by ∼59–91% compared to single isolated ribosomes (Supplementary Figure S3f). These results indicate that the *AAP_FLAG_* uORF occupancy duration of individual ribosomes was generally increased when ribosomes were clustered in the uORF, suggesting that ribosome crowding in the uORF caused slower movement of uORF-translating ribosomes.

### Ribosome stalling in the *AAP_FLAG_* uORF stimulates the uORF initiation efficiency

**Figure 4.**
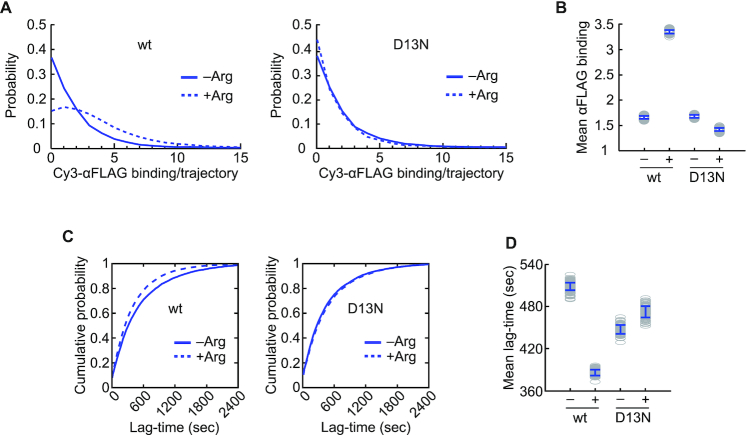
The AAP_FLAG_ response to Arg stimulates native-context *AAP_FLAG_* uORF initiation. (A, B) Probability distributions (**A**) and mean values (**B**) of Cy3-αFLAG binding number per trajectory. Mean ± SEM: 1.69 ± 0.02 (wt –Arg), 3.52 ± 0.03 (wt +Arg), 1.72 ± 0.02 (D13N –Arg), 1.42 ± 0.03 (D13N +Arg). The numbers of trajectories analyzed are the same as in Figure 2B, C. (C, D) Cumulative probabilities (**C**) and mean values (**D**) of the time-lags between the onsets of Cy3-αFLAG binding events. Mean ± SEM (s): 508 ± 6 (wt –Arg), 386 ± 3 (wt +Arg), 447 ± 6 (D13N –Arg), 472 ± 7 (D13N +Arg). Numbers of binding events analyzed: wt –Arg, *n* = 8002; wt +Arg, *n* = 25 582; D13N –Arg, *n* = 10 129; D13N +Arg, *n* = 4699.

To further assess the effects of AAP-mediated and Arg-induced ribosome stalling on uORF ribosome dynamics, we measured the amounts of Cy3-αFLAG binding per trajectory for *AAP_FLAG_-LUC_HA_* mRNA translation in the presence of either low or high Arg. High Arg increased the Cy3-αFLAG binding amount per trajectory by ∼2-fold for *WT-AAP_FLAG_-LUC_HA_* mRNA (mean binding number: 1.69 ± 0.02 [–Arg] versus 3.52 ± 0.03 [+Arg]) (Figure 4A, B), but did not have a significant effect with *D13N-AAP_FLAG_-LUC_HA_* mRNA (mean binding number: 1.72 ± 0.02 [–Arg] versus 1.42 ± 0.03 [+Arg]) (Figure 4A, B). For uORF initiation context-improved *AAP_FLAG_-LUC_HA_* mRNAs, high Arg also increased the amount of Cy3-αFLAG binding per trajectory with the wild-type uORF by approximately 1.5-fold (mean binding amount: 2.16 ± 0.03 [–Arg] versus 3.32 ± 0.02 [+Arg]) but not with the D13N uORF (1.86 ± 0.02 [–Arg] versus 1.96 ± 0.02 [+Arg]) (Supplementary Figure S4a,b). These observations indicate that the AAP_FLAG_ response to high Arg increased the number of ribosomes that translated the *AAP_FLAG_* uORF.

The increased translation of the wild-type, but not D13N, *AAP_FLAG_* uORF with high Arg (Figure 4A, B and Supplementary Figure S4a,b) indicated that the uORF initiation efficiency was increased by the AAP_FLAG_ response to high Arg. To quantify the uORF initiation rate, we measured the time-lags between the onsets of subsequent Cy3-αFLAG binding events. The results show that high Arg reduced the Cy3-αFLAG binding time-lag by 122 s (mean 508 ± 6 s [–Arg] versus 386 ± 3 s [+Arg]) for *WT-AAP_FLAG_-LUC_HA_* mRNA but did not have a significant impact for *D13N-AAP_FLAG_-LUC_HA_* mRNA (mean 447 ± 6 s [–Arg] versus 472 ± 7 s [+Arg]) (Figure 4C, D). Consistently, high Arg reduced the Cy3-αFLAG binding time-lag by 92 s (mean 495 ± 6 s [–Arg] versus 403 ± 2 s [+Arg]) with uORF initiation context-improved *WT-AAP_FLAG_-LUC_HA_* mRNA, but did not have a significant effect with context-improved *D13N-AAP_FLAG_-LUC_HA_* mRNA (mean 510 ± 5 s [–Arg] versus 487 ± 5 s [+Arg]) (Supplementary Figure S4c, d). Altogether, these data show that the wild-type AAP_FLAG_ response to high Arg stimulated the *AAP_FLAG_* uORF initiation efficiency.

### The uORF and mORF initiation efficiencies are anticorrelated on individual mRNAs

**Figure 5.**
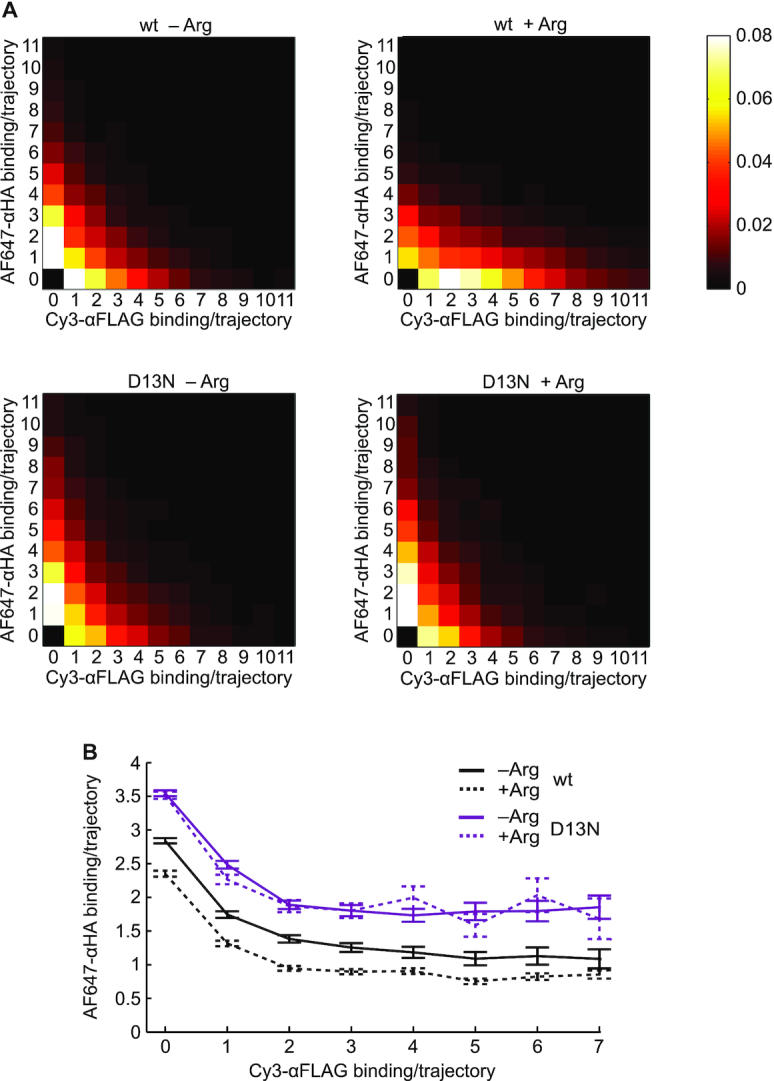
Smart-ORF detects anticorrelated uORF and mORF translation on individual native-context *AAP_FLAG_*-*LUC_HA_* mRNAs. (**A**) Heatmap representations of the number of uORF translation events (x-axis) vs. mORF translation events (y-axis) on individual native-context *AAP_FLAG_*-*LUC_HA_* mRNAs. The color-gradient of each box represents the probability of observing mRNA molecules with x number of Cy3-αFLAG binding events and y number of AF647-αHA binding events, as indicated by the scale bar. The numbers of trajectories analyzed are the same as in Figure 2B, C. (**B**) Mean numbers of AF647-αHA (y-axis) binding for mRNAs with x number of Cy3-αFLAG (x-axis) binding. The y-axis values were calculated as the mean values for each column of the heat map in (A).

To assess the impact of *AAP_FLAG_* uORF translation dynamics on the dynamics of *LUC_HA_* mORF translation, we measured the Cy3-αFLAG and AF647-αHA binding amounts per trajectory for individual *AAP_FLAG_-LUC_HA_* mRNAs and illustrated the correlation between these two quantities as heatmaps (Figure 5A). For all test conditions, uORF and mORF translation showed an anticorrelation, that is, mRNA molecules with larger numbers of Cy3-αFLAG binding tended to have smaller numbers of AF647-αHA binding. This suppression of mORF translation did not require the wild-type AAP_FLAG_ or its response to high Arg, indicating that the anticorrelated uORF and mORF translation reflected an intrinsic property of ribosome dynamics on single mRNA molecules. The heatmaps also show that high Arg extends the x-axis range with *WT-AAP_FLAG_-LUC_HA_* mRNA, but not with *D13N-AAP_FLAG_-LUC_HA_* mRNA, consistent with our observation of increased Cy3-αFLAG binding amount per mRNA caused by the AAP_FLAG_ response to high Arg (Figure 4A, B). Furthermore, among mRNA molecules with identical numbers of Cy3-αFLAG binding, *WT-AAP_FLAG_-LUC_HA_* mRNAs with high Arg showed significantly reduced AF647-αHA binding compared to all other conditions (Figure 5B). This effect is a likely consequence of the lengthened ribosome duration in the wild-type *AAP_FLAG_* uORF that results from the AAP_FLAG_ response to high Arg (Figure 3). Qualitatively similar results were also observed with context-improved *AAP_FLAG_-LUC_HA_* mRNAs (Supplementary Figure S5a, b). These results indicate that ribosome stalling in the *AAP_FLAG_* uORF augments an intrinsic ability of uORF translation to suppress mORF translation.

## DISCUSSION

Using a two-color single-molecule approach, we developed Smart-ORF to simultaneously track translation dynamics of individual ribosomes in both a uORF and mORF during active *in vitro* translation. We applied this method to translation of a bicistronic mRNA (*AAP_FLAG_-LUC_HA_*) with an AAP-encoding uORF to better understand the translational control mediated by this uORF-encoded ribosome arrest peptide. The *AAP_FLAG_* uORF was designed to encode a 42x CAA-repeat spacer region to allow N-terminal FLAG-tag exposure from the ribosome tunnel prior to translation of the C-terminal AAP. Alternative spacer sequences may also be suitable for the Smart-ORF assay but should be carefully chosen to ensure that they contain at least 35 codons and do not introduce cognate initiation sites, near-cognate initiation sites, or stable secondary structures. In the current setup, commercially available AF647-αHA allowed Smart-ORF quantification of mORF initiation efficiency and the correlation between uORF and mORF translation dynamics. However, fluorophore instability of the AF647 dye limited 80S dwell time accuracy for analysis of mORF translation. Measurement of 80S dwell time in a mORF can be achieved by using antibodies labeled with dyes of significantly greater photostability, such as triplet-state quencher-conjugated dyes (45). When high quality antibody/epitope pairs and photostable dyes are used, premature antibody dissociation, fluorophore photobleaching, and premature peptide release have negligible effects on ribosome dwell time measurements, as we demonstrated previously for Cy3-αFLAG (21). In such conditions, the measured ribosome dwell time is the combined durations of peptide elongation and peptide release and thereby corresponds to the duration of 80S in the uORF. To examine 80S dwell times on mRNAs with multiple uORFs, the Smart-ORF assay can be adapted to incorporate additional distinct epitope/antibody pairs into additional uORFs to allow the distinct uORFs to be examined either one at a time or simultaneously depending on the fluorescence imaging capability.

Previous *S. cerevisiae in vivo* studies demonstrated that AAP-mediated Arg-specific translational regulation is coupled to *CPA1* mRNA susceptibility to nonsense-mediated mRNA decay (NMD) (28), a eukaryotic surveillance mechanism that targets mRNAs undergoing premature translation termination for rapid degradation (46). NMD of *CPA1* mRNA was triggered by increased ribosomal occupancy of the uORF termination codon (28). However, studies with *S. cerevisiae* extracts from wild-type and NMD-defective cells indicate that synthetic mRNA is not destabilized by NMD in extract (28,47,48). Consistently, we did not observe evidence of NMD-mediated mRNA degradation in our experiments. However, it is possible that NMD factors participate in translational events in the Smart-ORF system, as described previously in cell- and extract-based systems (47,49–52).

uORF modulation of mORF initiation may occur by leaking scanning or reinitiation. The *CPA1* uORF start codon was predicted to be in a relatively poor context by calculations that evaluate initiation context favorability in *S. cerevisiae* (53). Consistent with this, previous toeprinting studies with *S. cerevisiae* extract demonstrated that the *CPA1* uORF inefficiently captures scanning ribosomes and that mORF initiation occurs even in presence of the elongation inhibitor, cycloheximide (43). Furthermore, improvement of the *CPA1* uORF initiation context in mRNAs containing a luciferase-encoding mORF caused reduced levels of luciferase activity in *S. cerevisiae* extract (43) and cells (28). Collectively, these results suggest that leaking scanning underlies AAP-mediated translational control. Consistent with these previous observations, we observed that improvement of the *AAP_FLAG_* uORF initiation context in *AAP_FLAG_-LUC_HA_* mRNA reduces luciferase activity in bulk reactions (Figure 2A) and reduces AF647-αHA binding per trajectory in the Smart-ORF system (Figure 2C, D). Leaky-scanning on *AAP_FLAG_-LUC_HA_* mRNA was therefore preserved and led to *LUC_HA_* mORF initiation in the Smart-ORF system.

Previous biochemical and structural studies support a mechanism in which the AAP response to Arg inhibits ribosome peptidyl transferase function and thereby arrests the movement of ribosomes at the uORF termination codon (12,13,29,30,42). While these studies are consistent with a stalling mechanism, regulated duration of individual ribosomes in a uORF had not been measured. Smart-ORF allowed us to track and quantify several aspects of uORF and mORF translation kinetics with single ribosome and single mRNA resolution, including initiation efficiency, 80S dwell time, polysome formation (see below), and the correlation between uORF and mORF translation dynamics. These capabilities of the Smart-ORF method enabled previously unattainable insights into the dynamics and regulation of 80S ribosomes mediated by the *CPA1* uORF.

Our Smart-ORF results indicate that uORF-encoded AAP-mediated responses to high Arg increase the median values of isolated ribosome dwells by ∼22% and ∼42% for native-context (Figure 3C) and improved-context (Supplementary Figure S3b) uORFs, respectively, which confirmed that the AAP_FLAG_ response to high Arg induced ribosome stalling in the *AAP_FLAG_* uORF. However, this extent of ribosome pausing in the uORF appeared insufficient to account for the ∼1.6-fold (native-context; Figure 2C) and ∼1.8-fold (improved-context; Figure 2D) reduction of *LUC_HA_* mORF translation, as measured by the number of AF647-αHA binding events per trajectory. Intriguingly, our kinetic observations revealed a distinct mechanism that led to a significantly greater extent of ribosome occupancy in the uORF. We found that the AAP-mediated response to high Arg stimulated the uORF initiation efficiency and increased the mean number of uORF-translating ribosomes per mRNA by ∼2.1-fold and ∼1.5-fold for native- (Figure 4B) and improved- (Supplementary Figure S4b) context uORFs, respectively. This translational regulation expands the total time of ribosome occupancy in the uORF.

Increased single ribosome dwells in the uORF and stimulated uORF initiation efficiency were only observed with high Arg when the uORF encoded the wild-type, but not the D13N, AAP_FLAG_ polypeptide, confirming that the above observations were Arg-dependent AAP-mediated effects. Considering that the AAP coding sequence is 210 nt away from the *AAP_FLAG_* uORF initiation codon, the nascent AAP appears too distant from the uORF start site to directly modulate uORF initiation efficiency. Furthermore, previous structural and toeprinting studies have verified that the AAP is a ribosome arrest peptide that interacts with the ribosome within the exit tunnel and increases ribosome occupancy at the uORF termination codon (13,29,42). These observations indicate that stimulated uORF initiation efficiency is a secondary effect of the AAP-mediated ribosome pause. Collectively, our data suggest a mechanism that links a significantly increased uORF initiation efficiency to a modestly increased duration of single ribosomes at the uORF termination codon.

We also observed clustered Cy3-αFLAG binding, indicating polysome formation in the *AAP_FLAG_* uORF. Polysomes form when two or more translating ribosomes are clustered on an mRNA (54–57). Our results show that the ratios of ribosomes involved in isolated vs. clustered binding were approximately 45% vs. 55% for the wild type AAP_FLAG_ and high Arg condition but were approximately 60% versus 40% for all other conditions (Supplementary Figure S6a, b). AAP-mediated ribosome stalling and stimulation of uORF initiation efficiency, therefore, appeared to cause a noticeable increase in the occurrence of ribosome clustering. The observed clustered binding was not an artifact of nonspecific Cy3-αFLAG binding because both the total number of binding events (Supplementary Figure S6a,b) and the proportion of binding events (Supplementary Figure S6c,d) in clusters for nonspecific binding were ∼300–1200-fold less and ∼2-fold less, respectively, than for specific binding. Therefore, less than ∼0.33% of clustered binding events could result from nonspecific binding. Interestingly, compared to the *AAP_FLAG_* uORF occupancy duration of single isolated ribosomes, the *AAP_FLAG_* uORF occupancy duration of individual ribosomes in clusters was prolonged by ∼1.6–2 fold, regardless of Arg concentration, D13N mutation, and uORF initiation context (Figure 3F and Supplementary Figure S3f). This general effect of clustered ribosomes may reflect translation-slowing inter-ribosomal interactions caused by the increased ribosome density in clusters (58–60). These data provide support for the idea that, as part of the AAP response to high Arg, ribosome crowding in the uORF exerts a layer of regulation that slows the movement of uORF-translating ribosomes.

**Figure 6.**
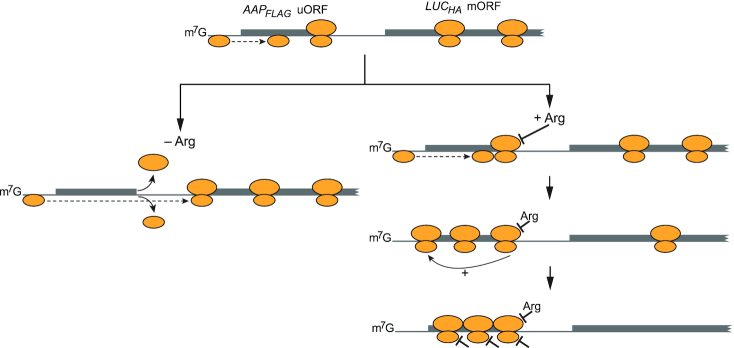
Model for ribosome dynamics on *AAP_FLAG_*-*LUC_HA_* mRNA. Left branch (–Arg condition): uORF initiation is inefficient and most 43S ribosomal particles scan past the uORF (dashed arrow). When infrequent uORF translation events occur, the 80S ribosomes translate efficiently and are quickly released from the uORF following termination (curved arrows). Therefore, subsequent scanning 43S ribosomal particles are not hindered by uORF translation and can efficiently access the downstream mORF. Right branch (+Arg condition): when infrequent uORF translation events occur, the nascent AAP briefly stalls uORF-translating 80S ribosomes at the uORF termination codon. The modest ∼22% increase in the duration of individual 80S ribosome dwells in the uORF (Figure 3A, B) temporarily impedes trailing scanning 43S ribosomal particles (dashed arrow), which stimulates the uORF initiation efficiency and increases the number of uORF initiation events by ∼2-fold (Figure 4). The increased ribosome loading onto the uORF leads to a distinct effect of ribosome crowding in the uORF, which further slows the translation speed of individual ribosomes by ∼2-fold (Figure 3F). The collective direct and secondary effects of ribosome stalling amplify ribosome occupancy of the uORF, which greatly suppresses mORF initiation (Figures 2 and 5).

Taken together with previous bulk studies, our results indicate that *CPA1* mORF translation is controlled through a dynamic and multi-layered mechanism involving interactions between the uORF-encoded nascent AAP, translating ribosomes, and scanning ribosomal particles in the 5′ leader of *CPA1* mRNA (Figure 6). When Arg concentrations are low, scanning 43S ribosomal particles predominantly scan through the *AAP_FLAG_* uORF without initiating. When the infrequent initiation event at the uORF does occur, ribosomes proceed at a normal pace while engaged in peptide elongation and termination and do not significantly block trailing scanning 43S. When Arg concentrations are high and an infrequent uORF initiation event occurs, the translating ribosome stalls at the termination codon through the AAP’s inhibition of PTC function, which delays termination and 80S dissociation. This increased duration of ribosomes at the uORF termination codon temporarily blocks trailing scanning 43S. The increased duration of impeded 43S in the uORF stimulates uORF initiation, possibly through oscillating 43S scanning activity (61) or ribosome queueing (62) in the mRNA leader. As newly initiated ribosomes are also subject to AAP-mediated stalling at the uORF termination codon, this mechanism could lead to multiple rounds of ribosome stalling and stimulated uORF initiation that potentiate clustering of uORF-translating ribosomes. When clustering occurs, the dynamics of individual translating ribosomes in the uORF are further reduced. Altogether, while AAP-mediated stalling exerts a relatively small direct effect on individual ribosome dynamics in the uORF, a chain reaction of subsequent translational events amplifies this effect into a significantly increased integration of multiple uORF-occupying ribosomes, consequently suppressing 43S scanning to the downstream mORF (Figure 6).

Our findings show that small perturbations in 80S translation can extensively modulate the ribosome traffic of translating 80S and scanning 43S on individual mRNA molecules. In addition to AAP-mediated regulation, ribosome traffic may also be dynamically modulated by a broad range of elements with ribosome pausing potential, including mRNA secondary structures (63), RNA-binding proteins (64), inhibitory codon pairs (65), rare codons (66), poly(A) tracts (65,67), chemically modified or damaged nucleotides (68), and drug-like molecules that directly bind and stall ribosomes (69). Although this study focused on AAP-mediated translational regulation, the Smart-ORF strategy presented here can be further extended to other *cis*- and *trans*-acting ribosome stalling elements. Adaption of this method may also facilitate efforts to understand the increasingly emerging connection between the local elongation rate and human health (70,71). Furthermore, similar to the one-color assay (21), the two-color assay can be combined with existing biochemical and genetic approaches to study factor functions. By generating factor-depleted extract and supplementing it with wild-type or mutant factor, single-molecule observations of both uORF and mORF 80S translation dynamics can identify factor functions that modulate ribosome traffic and regulate mORF translation. Mechanistic insights can be further expanded with the combined use of fluorescently labeled factors. The capabilities of Smart-ORF make this technology a powerful new tool to interrogate translational control mechanisms that modulate dynamics of uORF and mORF translation.

## Supplementary Material

gkaa1185_Supplemental_File
