# Supplementary material for: Smart-ORF: a single-molecule method for accessing ribosome dynamics in both upstream and main open reading frames
Source: Nucleic Acids Res. 2020 Dec 16;49(5):e26. doi: 10.1093/nar/gkaa1185 (PMC7969011; doi:10.1093/nar/gkaa1185)

## SUPPLEMENTAL FIGURE LEGENDS

**Supplementary Fig. 1 The Smart-ORF system. a**, Predicted minimum free energy (MFE) secondary structure of *AAP*$_{FLAG}$-*LUC*$_{HA}$ mRNA. The CAA repeat region in the *AAP*$_{FLAG}$ uORF is indicated. The confidence level of the predicted structure is indicated by color as defined in the colormap scale bar. The MFE structure was computed using RNAfold 2.4.11 from the ViennaRNA Webserver(1). **b**, The sequence of 5′ leader regions of *AAP*$_{FLAG}$-*LUC*$_{HA}$ genes. The sequence begins with the T7 RNA polymerase-binding site and ends within the HA coding region. Firefly luciferase coding sequence downstream of the HA coding region is not shown. *AAP*$_{FLAG}$ uORF and *LUC*$_{HA}$ mORF sequences are shown in bold. The *CPA1* AAP amino acid sequence is shown above the corresponding DNA sequence. The improved uORF initiation context and D13N mutations are shown below the wild-type sequence. **c**, Fig. 1e is shown using a linear scale in the x-axis.

**Supplementary Fig. 2 Probability density distributions of Cy3-αFLAG binding dwell times for *AAP*$_{FLAG}$-*LUC*$_{HA}$ mRNAs.** Single (**a**,**b**) and clustered (**c**,**d**) Cy3-αFLAG binding events. The cumulative probability distributions for the dwell time analyses described in Fig. 3a–d (native context) and Supplementary Fig. 3a–d (improved context) are shown here as probability density distributions.

**Supplementary Fig. 3 Smart-ORF resolves Arg-induced ribosome dwells in the initiation context-improved *AAP*$_{FLAG}$ uORF. a**–**d**, The analyses described in Fig. 3b–e but for initiation context-improved *AAP*$_{FLAG}$-*LUC*$_{HA}$ mRNAs. **a**,**b**, Cy3-αFLAG dwell times for single binding events. Median and S.E. (sec): 60±1 (↑wt –Arg), 85±1 (↑wt +Arg), 70±1 (↑D13N –Arg), 66±1 (↑D13N +Arg). Numbers of single binding events analyzed: ↑wt –Arg, n = 6690; ↑wt +Arg, n = 24,699; ↑D13N –Arg, n = 9524; ↑D13N +Arg, n = 10,121. **c**,**d**, Cy3-αFLAG dwell times for clustered binding events. Median value and S.E. (sec): 153±6 (↑wt –Arg), 304±5 (↑wt +Arg), 224±9 (↑D13N –Arg), 189±5 (↑D13N +Arg). Numbers of binding clusters analyzed: ↑wt –Arg, n = 1687; ↑wt +Arg, n = 9801; ↑D13N –Arg, n = 2363; ↑D13N +Arg, n = 2678. **e**, Dwell time analysis of isolated Cy3-αFLAG binding events for native-context *AAP*$_{FLAG}$-*LUC*$_{HA}$ mRNAs using intensity integration- (left panel) or threshold- (right panel, the same as in Fig. 3b) based approaches (Materials and Methods) yields similar trends between the different translation conditions. Intensity integration approach median and S.E. (sec): 61±1 (wt –Arg), 67±1 (wt +Arg), 59±1 (D13N –Arg), 57±2 (D13N +Arg). Threshold approach median and S.E. are the same as in Fig. 3b,c. **f**, The analysis described in Fig. 3f but for initiation context-improved *AAP*$_{FLAG}$-*LUC*$_{HA}$ mRNAs. Isolated binding median dwell and S.E. (sec): 55±1 (↑wt –Arg), 73±1 (↑wt +Arg), 65±1 (↑D13N –Arg), 63±1 (↑D13N +Arg). In cluster binding median dwell and S.E. (sec): 87±4 (↑wt –Arg), 140±2 (↑wt +Arg), 108±3 (↑D13N –Arg), 101±3 (↑D13N +Arg). The numbers of binding events analyzed are the same as in **a**–**d**.

**Supplementary Fig. 4 The AAP$_{FLAG}$ response to Arg stimulates context-improved *AAP*$_{FLAG}$ uORF initiation.** The analyses described in Fig. 4 but for initiation context-improved *AAP*$_{FLAG}$-*LUC*$_{HA}$ mRNAs. Mean ± SEM for **b**: 2.16±0.03 (↑wt –Arg), 3.32±0.02 (↑wt +Arg), 1.86±0.02 (↑D13N –Arg), 1.96±0.02 (↑D13N +Arg). Number of trajectories analyzed for **a**,**b**: ↑wt –Arg, n = 5747; ↑wt +Arg, n = 16,658; ↑D13N –Arg, n = 9672; ↑D13N +Arg, n = 9821. Mean ± SEM (sec) for **d**: 495±6 (wt –Arg), 403±2 (wt +Arg), 510±5 (D13N –Arg), 488±5 (D13N +Arg). Number of binding events analyzed for **c**,**d**: ↑wt –Arg, n = 7907; ↑wt +Arg, n = 40,539; ↑D13N –Arg, n = 10,909; ↑D13N +Arg, n = 12,079.

**Supplementary Fig. 5 Smart-ORF detects anticorrelated uORF and mORF translation on individual improved-context *$AAP_{FLAG}$-$LUC_{HA}$* mRNAs.** Analysis as described in Fig. 5 but for initiation context-improved *$AAP_{FLAG}$-$LUC_{HA}$* mRNAs. The numbers of trajectories analyzed are the same as in Fig. 2d,e.

**Supplementary Fig. 6 Ribosome stalling promotes ribosome clustering in the *$AAP_{FLAG}$* uORF. a, b**: The mean and s.e. of the numbers of single binding events (blue) and binding events in clusters (cyan) per data set are shown for nonspecific (NS) and specific Cy3-αFLAG binding with the native (**a**) and improved (**b**) uORF initiation contexts. Numbers of data sets analyzed: NS, n = 1; wt –Arg, n = 3; wt +Arg, n = 3; D13N –Arg, n = 3; D13N +Arg, n = 2; ↑wt –Arg, n = 5; ↑wt +Arg, n = 5; ↑D13N –Arg, n = 3; ↑D13N +Arg, n = 3. **c, d**: Probability distributions of Cy3-αFLAG binding events for different cluster sizes with native- (**c**) and improved- (**d**) context *$AAP_{FLAG}$* uORFs. For simplicity of this analysis, single isolated translation events are represented as one ribosome binding "cluster". The probability distribution for nonspecific binding is shown in red for reference. Numbers of clusters analyzed with the native-context *$AAP_{FLAG}$* uORF in **c**: wt –Arg, n = 6452; wt +Arg, n = 17,003; D13N –Arg, n = 8231; D13N +Arg, n = 4176. Numbers of clusters analyzed with the improved-context *$AAP_{FLAG}$* uORF in **d**: ↑wt –Arg, n = 7694; ↑wt +Arg, n = 32,726; ↑D13N –Arg, n = 10,572; ↑D13N +Arg, n = 11,457. The number of clusters analyzed was 28 for the nonspecific binding condition.

## Reference

1. Lorenz, R., Bernhart, S.H., Honer Zu Siederdissen, C., Tafer, H., Flamm, C., Stadler, P.F. and Hofacker, I.L. (2011) ViennaRNA Package 2.0. *Algorithms Mol Biol*, **6**, 26.

# Supplementary figure 1

**a**

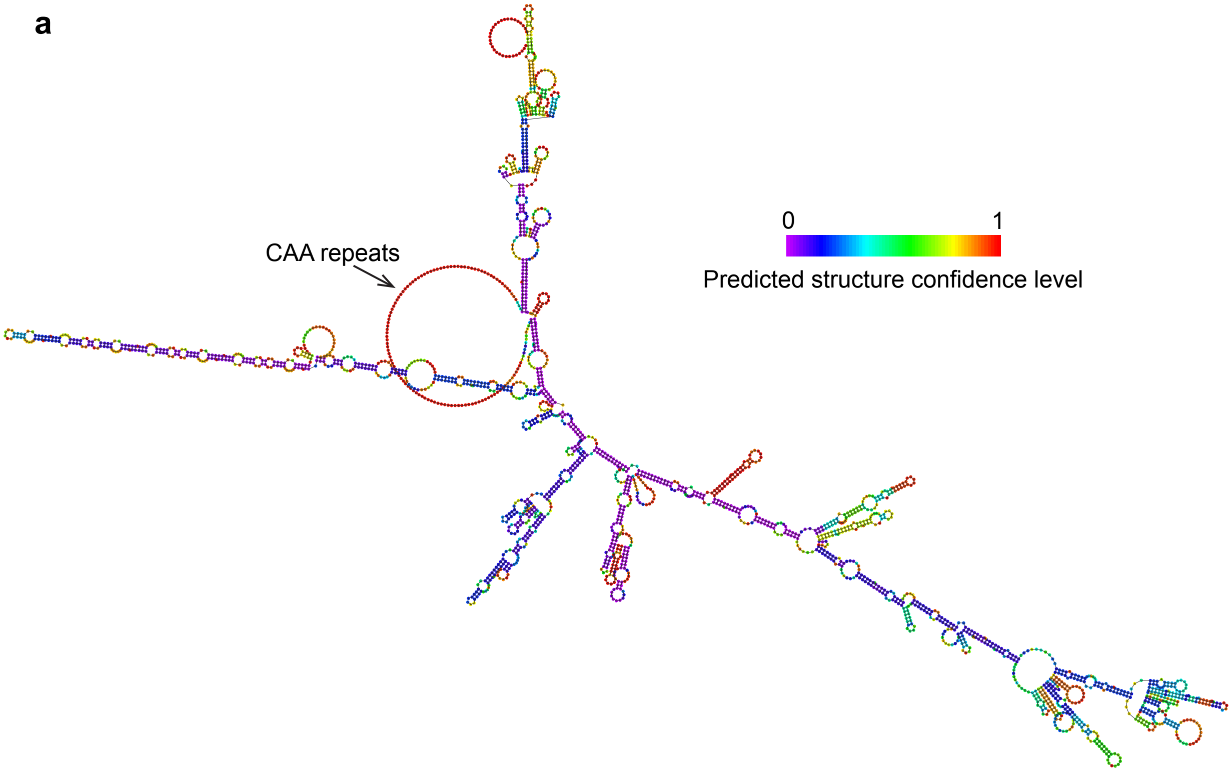


**b**

AAP$_{FLAG}$

...TAATACGACTCACTATAGATCTACCCTTTTTGCAGATTTGAAATAAAAAAAACATTAT**ATGTTCGACTACAA**
TTGAAAA
(improved context)

**AGACCACGACGGAGATTATAAAGATCACGATATCGATTACAAGGACGACGACGACAAGAAGCTTCAACAACAACA**

**ACAACAACAACAACAACAACAACAACAACAACAACAACAACAACAACAACAACAACAACAACAACAACAACAACA**

F S L S N S Q Y T C
**ACAACAACAACAACAACAACAACAACAACAACAACAACAAGGTACCTTTAGCTTATCGAACTCTCAATACACCTG**

Q D Y I S D H I W K T S S H *
**CCAAGACTACATATCTGACCACATCTGGAAAACTAGCTCCCACTAA**TTTCATTGCTTAATAATCAGAAATTCTAT
AAC
(D13N)

LUC$_{HA}$

CACAAACCACTCCTAAAAATATTTCAACC**ATGGCTTACCCATACGATGTTCCAGATTACGCTGGTTACCCAT...**

**c**

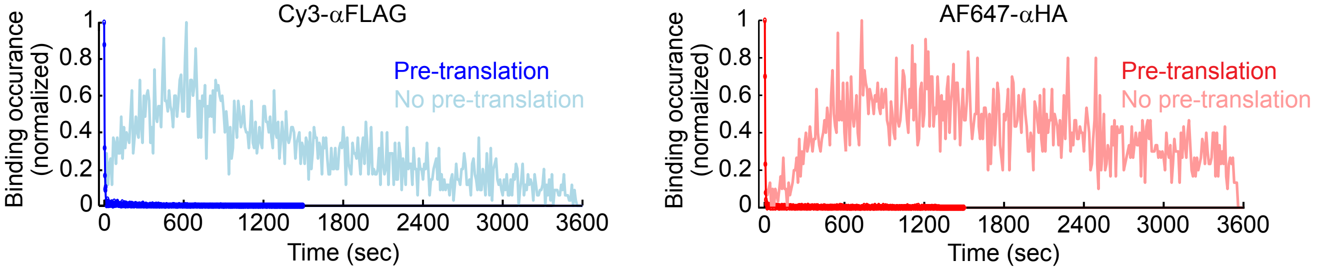

# Supplementary figure 2

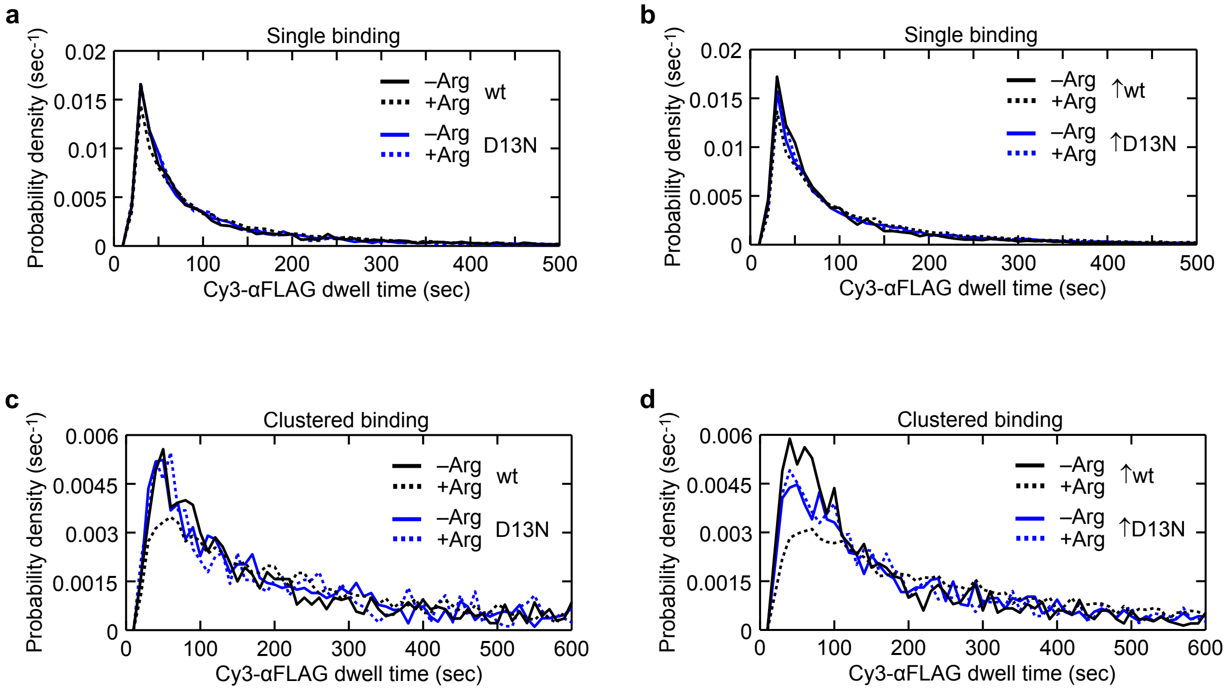

# Supplementary figure 3

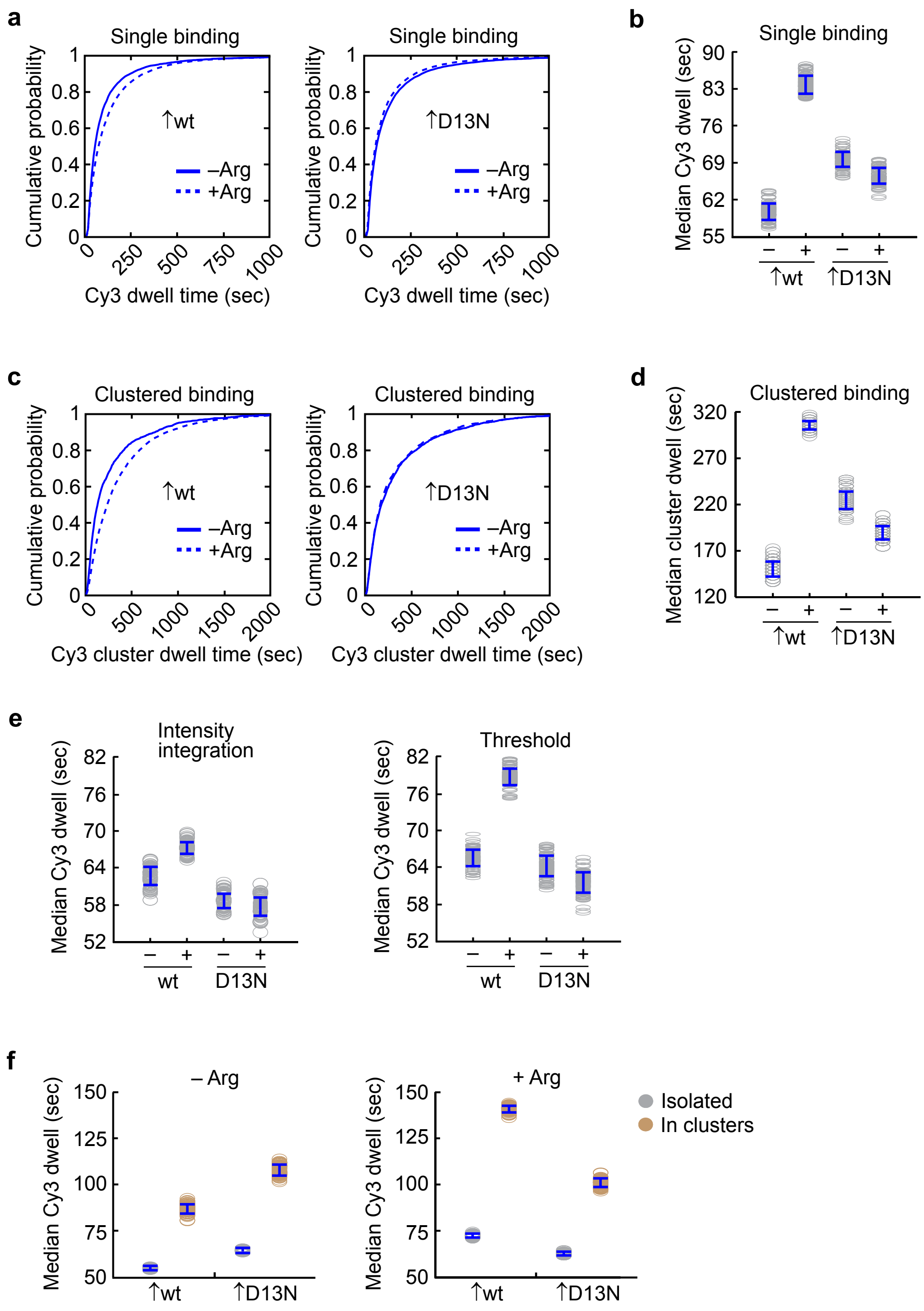

# Supplementary figure 4

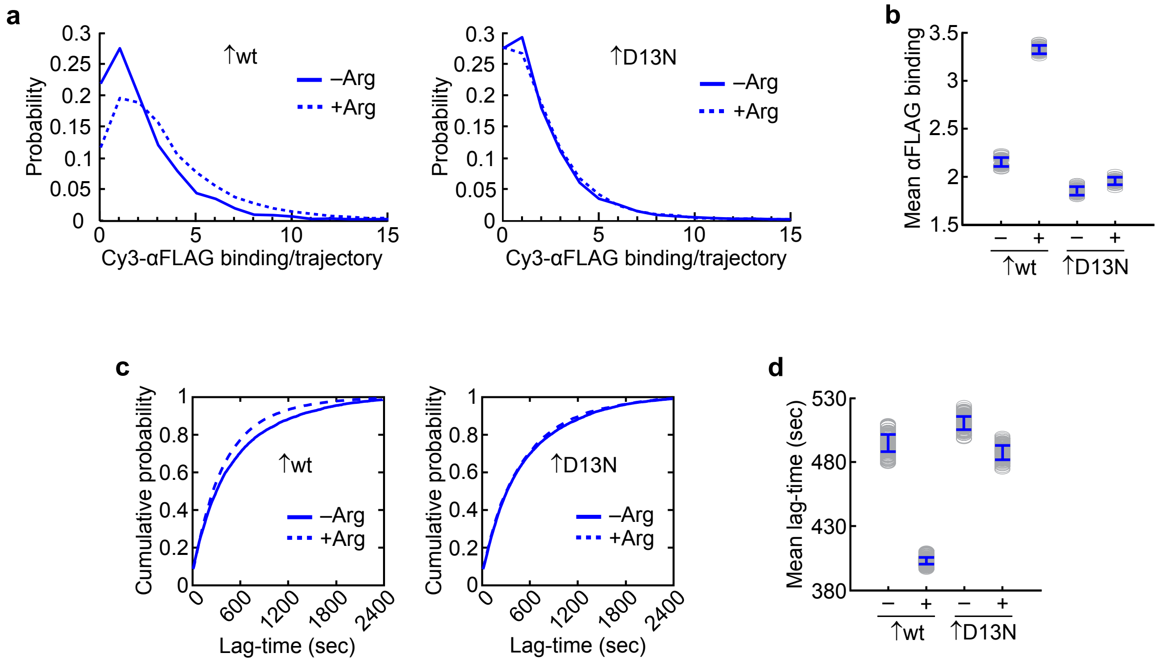

# Supplementary figure 5

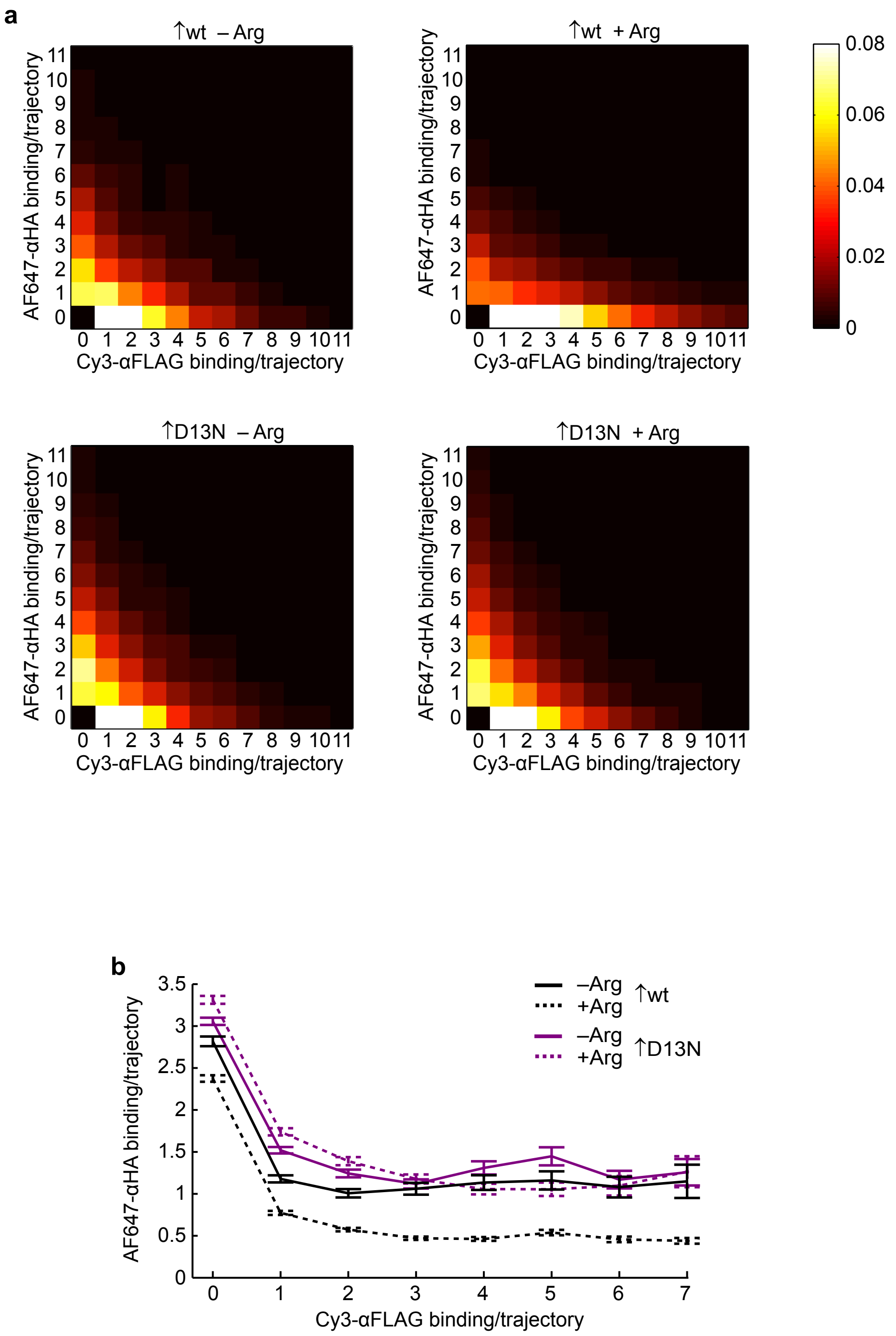

# Supplementary figure 6

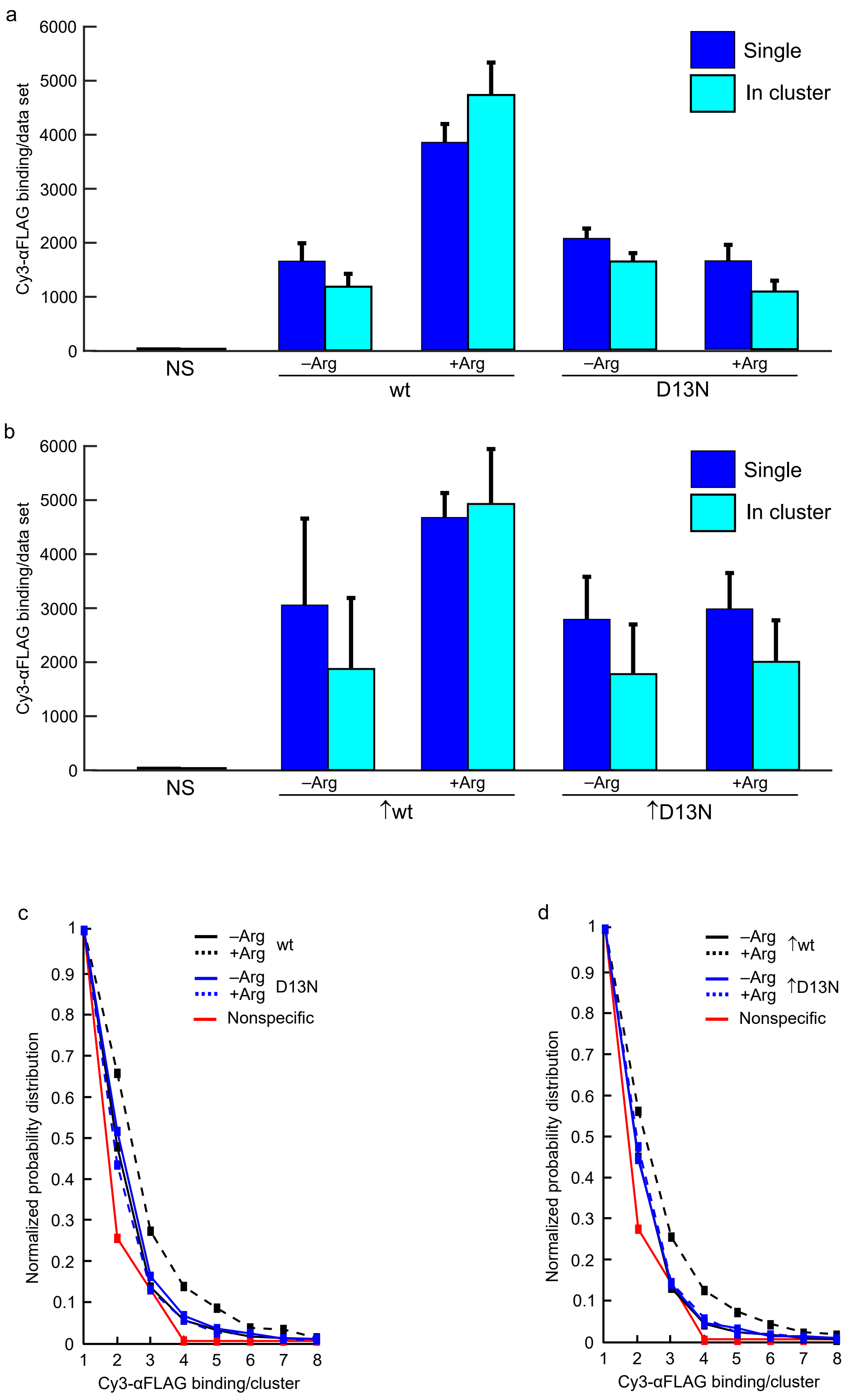

Supplement: gkaa1185_Supplemental_File [file gkaa1185_supplemental_file.pdf]
